# Development of an invasion score based on metastasis-related pathway activity profiles for identifying invasive molecular subtypes of lung adenocarcinoma

**DOI:** 10.1038/s41598-024-51681-9

**Published:** 2024-01-19

**Authors:** Tao Han, Yafeng Liu, Jiawei Zhou, Jianqiang Guo, Yingru Xing, Jun Xie, Ying Bai, Jing Wu, Dong Hu

**Affiliations:** 1 School of Medicine, Anhui University of Science and Technology, Chongren Building, No 168, Taifeng St, Huainan, 232001 China; 2 Anhui Province Engineering Laboratory of Occupational Health and Safety, Anhui University of Science and Technology, Huainan, 232001 China; 3 Key Laboratory of Industrial Dust Deep Reduction and Occupational Health and Safety of Anhui Higher Education Institutes, Anhui University of Science and Technology, Huainan, 232001 China; 4 Affiliated Cancer Hospital, Anhui University of Science and Technology, Huainan, 232035 China; 5 Department of Clinical Laboratory, Anhui Zhongke Gengjiu Hospital, Hefei, China

**Keywords:** Cancer, Computational biology and bioinformatics

## Abstract

The invasive capacity of lung adenocarcinoma (LUAD) is an important factor influencing patients’ metastatic status and survival outcomes. However, there is still a lack of suitable biomarkers to evaluate tumor invasiveness. LUAD molecular subtypes were identified by unsupervised consistent clustering of LUAD. The differences in prognosis, tumor microenvironment (TME), and mutation were assessed among different subtypes. After that, the invasion-related gene score (IRGS) was constructed by genetic differential analysis, WGCNA analysis, and LASSO analysis, then we evaluated the relationship between IRGS and invasive characteristics, TME, and prognosis. The predictive ability of the IRGS was verified by in vitro experiments. Next, the “oncoPredict” R package and CMap were used to assess the potential value of IRGS in drug therapy. The results showed that LUAD was clustered into two molecular subtypes. And the C1 subtype exhibited a worse prognosis, higher stemness enrichment activity, less immune infiltration, and higher mutation frequency. Subsequently, IRGS developed based on molecular subtypes demonstrated a strong association with malignant characteristics such as invasive features, higher stemness scores, less immune infiltration, and worse survival. In vitro experiments showed that the higher IRGS LUAD cell had a stronger invasive capacity than the lower IRGS LUAD cell. Predictive analysis based on the “oncoPredict” R package showed that the high IRGS group was more sensitive to docetaxel, erlotinib, paclitaxel, and gefitinib. Among them, in vitro experiments verified the greater killing effect of paclitaxel on high IRGS cell lines. In addition, CMap showed that purvalanol-a, angiogenesis-inhibitor, and masitinib have potential therapeutic effects in the high IRGS group. In summary we identified and analyzed the molecular subtypes associated with the invasiveness of LUAD and developed IRGS that can efficiently predict the prognosis and invasive ability of the tumor. IRGS may be able to facilitate the precision treatment of LUAD to some extent.

## Introduction

Lung cancer is the leading cause of cancer-related deaths worldwide, and its incidence rate is ranked second in the world^1^. Lung adenocarcinoma (LUAD) is the most common subtype of lung cancer which accounts for about 40% of all lung cancers^2–4^. Despite the great advances in cancer treatment in the fields of surgery, chemotherapy, radiotherapy, and targeted therapy in recent years have led to improved survival rates for LUAD^5^. However, there are still many LUAD patients who cannot achieve the desired outcome with conventional therapies due to the heterogeneity, metastasis, and drug resistance, and the heterogeneity within the tumor also leads to different benefit levels for each LUAD^6–9^. Therefore, it is necessary to develop a biomarker that can effectively differentiate different subtypes of LUAD for precise clinical treatment of patients to improve the prognosis of lung adenocarcinoma.

The invasive ability and metastatic ability of tumors are closely interrelated, and they directly affect patient prognosis as major hallmarks of cancer^10^. Accumulating evidence shows that molecular characteristics are generally altered between tumors with different invasiveness or between the primary and metastatic sites of tumors^11–13^. A number of studies have been performed to assess the malignancy of tumors by analyzing and identifying the gene expression patterns present within metastatic or aggressive tumors. For example, van’t Veer et al. used supervised classification to identify gene expression signatures that could efficiently distinguish whether breast cancer patients had metastases in their study^14^. Lin et al. developed a 17-gene signature for brain metastasis by analyzing the variation of the mRNA expression levels between brain metastatic tumors and primary tumors in LUAD and pointed out that the CDKN2A/p16 gene may have an important role in promoting brain metastasis in LUAD^15^. In addition, Yoo et al. identified an invasiveness signature consisting of 1322 genes by comparing transcriptome level differences between lymph node nonmetastatic and lymph node metastatic patients with early-stage lung adenocarcinoma^16^. There is no doubt that these studies can enhance the clinical diagnostic ability and the effect of treatment for metastatic and invasive tumors. However, most of the relevant studies are based on the molecular characteristics of metastatic and non-metastatic samples for comparison, which is likely to ignore the bias in the analysis results caused by some primary tumors that have a clear tendency to metastasize but have not yet metastasized.

Therefore, this study attempted to comprehensively characterize the invasive and metastatic ability of tumors by revealing the altered activity of metastasis-related pathways within tumors and analyzing the potential molecular heterogeneity between different invasive phenotypes. Subsequently, we constructed an invasion score to quantify the invasive ability of LUAD based on the molecular characteristics of the invasive subtypes, which may provide a possible reference for clinical precision therapy.

## Materials and methods

### Downloading and preprocessing of data

Transcriptomic data of TCGA-LUAD patients and clinical information were downloaded from the UCSC website (https://xenabrowser.net/datapages/) (there is 517 tumor samples and 59 normal samples. Among them, 502 patients have survival information), and mutation information of LUAD patients was obtained from The Cancer Genome Atlas (TCGA) database. Gene expression data and clinical information of GSE31210, GSE50081, GSE72094, GSE42127, GSE166722, GSE27717, GSE202859 and GSE136935 datasets were downloaded from the GEO database (https://www.ncbi.nlm.nih.gov/geo/). Download expression profiling data of NSCLC cell lines from the CCLE database (https://sites.broadinstitute.org/ccle/) (Supplementary Table 1). Using Metastasis as a keyword, C2—curated gene sets, and Homo sapiens as filters, 114 metastasis-associated gene sets were downloaded from the MSigDB website (http://www.gseamsigdb.org/gsea/msigdb/index.jsp) ( Supplementary Table 2), of which 110 gene sets were calculated as metastatic activity scores using gene set variation analysis (GSVA) (Supplementary Table 3), which can be used to detect small pathway activity changes within the entire gene expression set^17^. (The remaining 4 gene sets could not be calculated due to the insufficient number of genes (n < 5) contained in the TCGA-LUAD expression profile).

### Identification of invasive lung adenocarcinoma subtypes

Based on the metastatic activity score, unsupervised consistent clustering was performed on the LUAD patients using the R package “ConsensusClusterPlus”^18^, and K-mean (km) clustering Euclidean metric was used for analysis. Principal Component Methods (PCA) were used to detect transcriptional patterns in different subtypes. Kaplan–Meier (K–M) survival curves were used to analyze the survival differences between different subtypes of LUAD patients. Subsequently, genetic differential analysis was performed for different subtypes to obtain |log_2_FC| > 0.5, FDR < 0.05 differential genes were overlaid with known proliferation-related genes, metastasis-related genes, and invasion-related genes to visualize the distribution of different genetic features in different groups of patients in the form of radar plots. PROGENy algorithm was used to analyze the oncogenic pathway activity of patients^19^.

### Exploration of the tumor microenvironment

Twenty six stemness gene sets were obtained from a web-based tool, StemChecker (http://StemChecker.sysbiolab.eu/)^20^. ssGSEA quantitatively elucidated the stemness enrichment score of the 26 stemness gene sets in each LUAD sample. The ESTIMATE algorithm was used to calculate the immune score, stromal score and ESTIMATE score for each patient^21^. The TME subtypes (immune-enriched, fibrotic (IE/F), immune-enriched, nonfibrotic (IE); fibrotic (F); immune-depleted (D))^22^ and the immune subtype (Wound Healing (C1), IFN-γ Dominant (C2), Inflammatory(C3), Lymphocyte Depleted(C4), TGF-β Dominant (C6))^23^ information was obtained from the previous literature. The TIMER algorithm was used to calculate the abundance of immune cell infiltration for LUAD patients^24^. Waterfall plots were used to visualize the frequency of mutations in the top 20 ranked genes in different subtypes. This was followed by further analysis of the differences in the number of neoantigens produced in different subtypes, which was obtained from The Cancer Immunome Atlas (TCIA, https://tcia.at/home). In addition, we obtained immunosuppression-related genes from the TISIDB database (http://cis.hku.hk/TISIDB/). Stemness markers associated with lung cancer were summarized from the previous studies, including CD166, CD24, CD44, CD87, CD133 and CD90^25,26^.

### Establishment and validation of invasion-related gene scores (IRGS)

The “limma” package was used for differential analysis of normal and tumor samples in TCGA-LUAD, and the differential genes (n = 4462) were filtered with the criteria of |log_2_FC| > 1 and FDR < 0.05. The screened genes were subjected to weighted gene co-expression network (WGCNA) analysis, and the genes were divided into different functional modules and correlated with invasive lung adenocarcinoma subtypes to screen the key gene modules. Genes within the key modules were extracted using Module Membership (MM) > 0.8 and Gene Significance (GS) > 0.6 as criteria. Afterward, 15 genes were screened for prognostic signature using Least absolute shrinkage and selection operator (LASSO) regression analysis, and the regression coefficients β for each gene were calculated using the sum of the products of the expression levels of each gene and the regression coefficients β. The Kaplan–Meier (K–M) survival analysis was performed to compare the overall survival (OS), progression-free interval (PFI), and progression-free survival (PFS) differences between the high- and low-risk groups in the TCGA and GEO datasets.

### Functional enrichment analysis

GSEA was used to analyze the biological pathways enriched in patients with high risk. Gene set enrichment was considered significant only when the FDR was < 0.05.

### Cell culture and reagents

Human lung adenocarcinoma cell lines PC9, A549 and H1975 were purchased from Biochemistry and Cell Biology of the Chinese Academy of Sciences (Shanghai, China). The A549 and H1975 were cultured in DMEM medium, Gibco; PC9 is cultured in RPMI 1640 medium, Gibco; these mediums containing 10% fetal bovine serum (FBS; Lonsera) and 1% penicillin/streptomycin (Beyotime; C0222) and placed in a 37 °C, 5% CO2 incubator. In addition, paclitaxel was purchased from yuanye bio-technology company.

### Scratching experiment

LUAD cells at the logarithmic growth stage were collected, inoculated in cell culture dishes, and cultured to 90% fusion. The cells were scratched straight with a 200 μl sterile tip; the cells were washed twice with sterile PBS to remove the detached cells, serum-free medium was added, the culture was continued and images were taken at 24 h.

### Transwell migration assay

For migration assay, LUAD cells were collected at the logarithmic growth stage, and the cell density was adjusted to 2 × 10^5^ cells/ml with a serum-free medium. 200 μl of cell suspension was inoculated in the upper chamber, and 700 μl of 10% serum medium was added to the lower chamber. 24 h incubation at 37 °C was followed by removal of the small chamber, rinsing with PBS 3 times, fixing with 4% paraformaldehyde, and staining with 0.1% crystal violet. The visual fields were photographed by microscope and counted by ImageJ software. The operation of the invasion experiment is basically the same as migration, but in the invasion experiment, the Transwell chambers need to be wrapped with matrix gel before the experiment is performed.

### Drug sensitivity and drug screening

“oncoPredict” is an R package that predicts drug sensitivity based on gene expression. The “oncoPredict” R package based on the Genomics of Drug Sensitivity in Cancer (GDSC) (https://www.cancerrxgene.org/) was used to predict drug sensitivity by calculating the half-maximal inhibitory concentration (IC_50_) values for the commonly used drugs in each LUAD sample. And they were divided into a high IRGS group (n = 251) and a low IRGS group (n = 251) using the median value of IRGS scores as a cutoff, after which the IC_50_ values of the patients in the different groups were compared. Connectivity Map (CMap, https://clue.io/) is a database developed by the Broad Institute based on interfering with target gene expression to screen small molecule drugs. Potential drugs that promote or inhibit biological processes in tumors can be screened by up- and down-regulated gene expression profiles.

### Cell counting/MTS assay

We inoculated lung adenocarcinoma cells in 96-well plates according to 3 × 10^3^ per well overnight. After that, we added 2 μM paclitaxel respectively and incubated for 24 h, 48 h and 72 h. After that, MTS reagent (Promega) was added and incubated for 4 h and OD values were determined.

### Statistical analysis

Statistical analyses were performed using R software and GraphPad. The Student’s t-test or Wilcoxon test was used for pairwise comparisons between two groups and the Kruskal–Wallis test for multiple comparisons. Kaplan–Meier method is used for survival analysis and the chi-square test was used to compare categorical variables. p-value < 0.05 was considered significant.

## Results

### Identification of invasive subtypes based on metastasis-related pathway activity in lung adenocarcinoma

**Figure 1:**
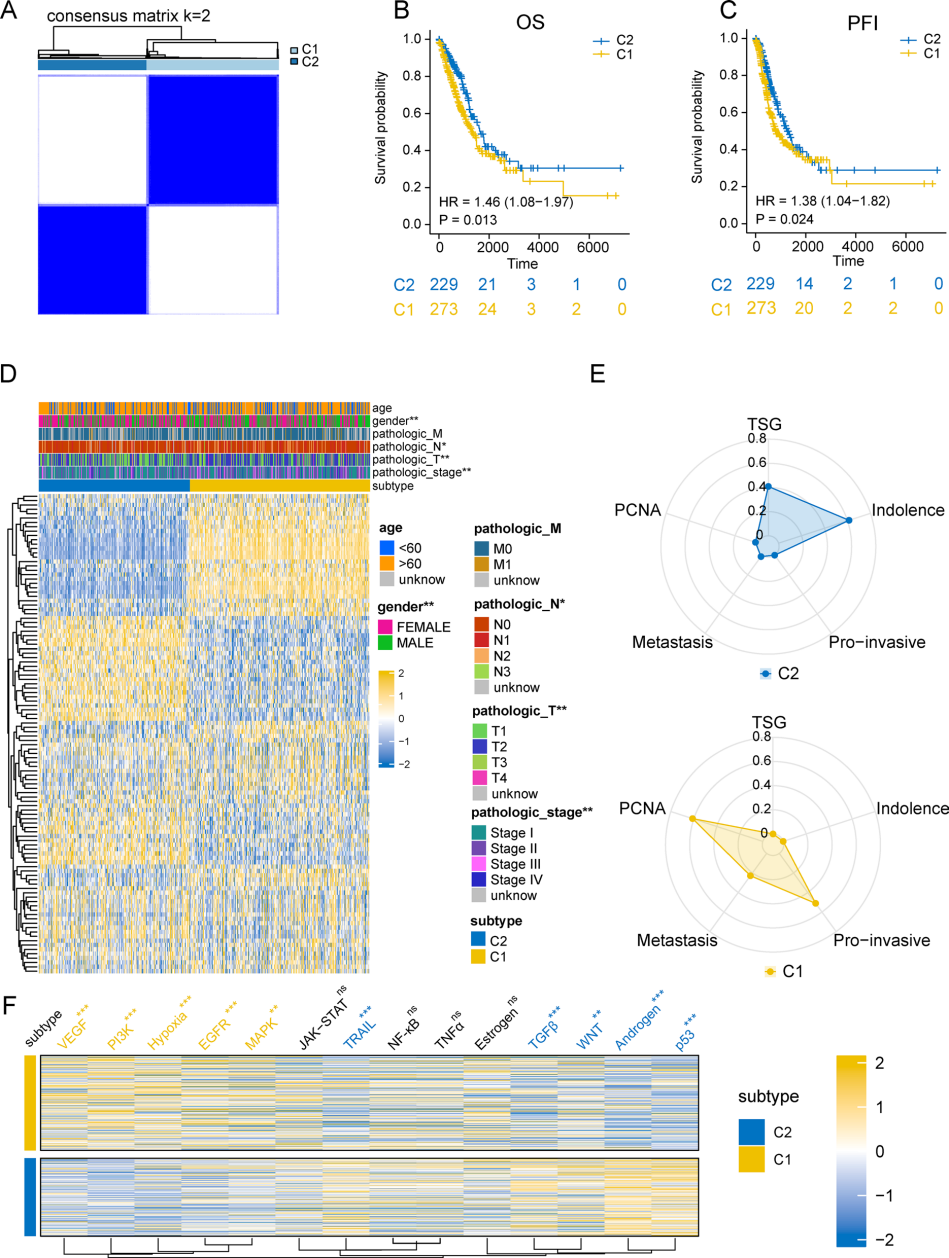
Identification of invasive subtypes (**A**) Unsupervised consistent clustering of patients with lung adenocarcinoma. (**B**,**C**) Differences in overall survival (OS) and progression-free interval (PFI) in patients with different subtypes. (**D**) Heatmap of the distribution of metastatic activity scores and clinicopathological features in patients with different subtypes. (**E**) Radar plot showing the distribution of different signature genes in C1 versus C2 groups. (**F**) The oncogenic signaling pathways activity scores was measured by PROGENy algorithm. (***p < 0.001; **p < 0.01; *p < 0.05; *ns* not significant).

The flowchart is shown in Supplementary Fig. 1. Herein, we first clustered LUAD patients based on metastasis-related pathway activity. The clustering results showed that the optimal number of clusters was obtained when K = 2, and LUAD patients were distinguished into C1 and C2 subtypes (Fig. 1A, Supplementary Fig. 2A,B). The principal component analysis (PCA) showed that the samples of these two subtypes were highly separated from each other (Supplementary Fig. 2C). K–M survival analysis showed that the overall survival (OS) and progression-free interval (PFI) of patients in the C2 subtype were significantly better than those in the C1 subtype (Fig. 1B,C). The heatmap showed the distribution of metastatic activity scores in different subtypes, while the distribution of clinical pathological features in LUAD subtypes showed that male patients were predominant in the C1 subtype, and the T-stage, N-stage and pathological stage were significantly higher than those in the C2 subtype (Fig. 1D). To further analyze the potential molecular differences between C1 and C2 subtypes, we obtained the genes upregulated in each of the two subtypes by genetic differential analysis (Supplementary Fig. 2D), and overlapped the upregulated genes with the known signature genes. The results showed that proliferation-associated Meta-PCNA signature genes^27^, Pro-invasive signature genes^16^ and metastasis signature genes^28^ were mainly enriched in the C1 subtype, while indolent signature genes^16^ and tumor suppressor genes (TSGene2.0, https://bioinfo.uth.edu/TSGene/)^29^ were mainly enriched in C2 subtype (Fig. 1E). Consistently, GSEA analysis showed that epithelial-mesenchymal transition and metastasis-related pathways were upregulated in the C1 group (Supplementary Fig. 2E). We then evaluated the malignant signaling pathway activity in different subtypes of patients using the PROGENy algorithm, which showed that VEGF, PI3K, Hypoxia, EGFR, and MAPK pathways were enriched in the C1 subtype (Fig. 1F).

### Heterogeneity of the tumor microenvironment between invasive subtypes in lung adenocarcinoma

**Figure 2:**
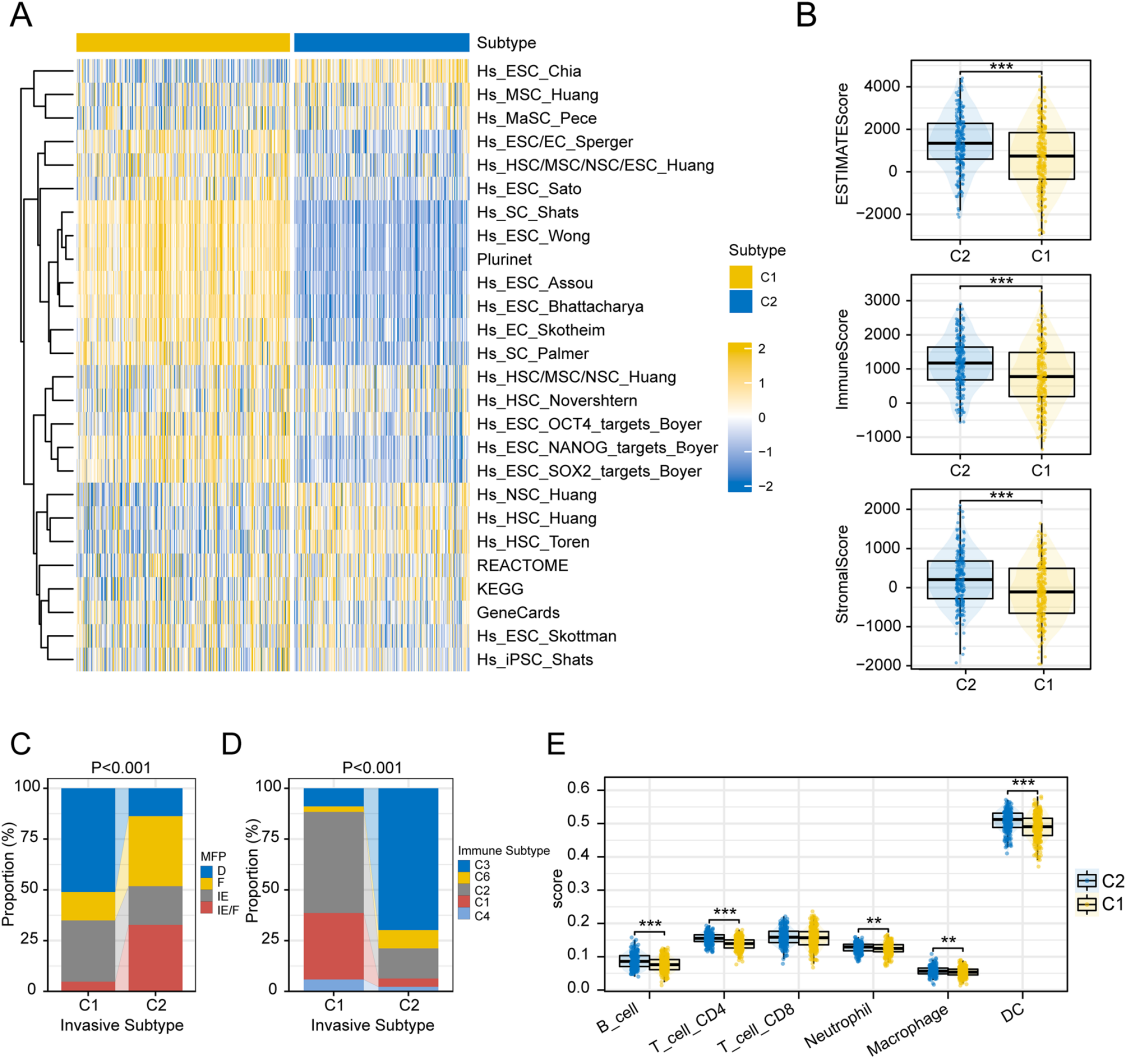
Differences in TME between two subtypes. (**A**) The activity of 26 stemness signatures between different invasive subtypes. (**B**) The distribution of stromal score, immune score, and ESTIMATE scores between different subtypes. (**C**,**D**) Differences in the distribution of patients with different invasive subtypes among defined TME subtypes and immune subtypes (Chi-square test). (**E**) TIMER algorithm to calculate the level of immune infiltration in patients with different subtypes. (***p < 0.001; **p < 0.01; *p < 0.05; *ns* not significant).

Current studies suggest that the metastatic ability of tumors is inextricably linked to the heterogeneity of the intra-tumoral microenvironment^30^. Cancer stem cells (CSC), as one of the components of the tumor microenvironment, have been suggested to be a key factor influencing tumor metastasis^31^. Here, we first evaluated the distribution of 26 stemness signature scores among two subtypes, and the results showed that most of them were significantly upregulated in the C1 subtype (Fig. 2A). In the term of immune infiltration, the ESTIMATE algorithm showed that the stromal score, immune score, and ESTIMATE score were all higher in C2 subtype than in the C1 subtype (Fig. 2B). Further deciphering the TME showed that among the four TMEs subtypes defined by Bagaev et al., “immune-depleted (D)” subtype was predominantly enriched in patients with C1 subtype and “immune-enriched, fibrotic (IE/F)” subtype was predominantly enriched in patients with C2 subtype (Fig. 2C). In terms of immune subtypes defined by Thorsson et al., the “Inflammatory subtype (C3)” was significantly enriched in the indolent C2 subtype. While, the “Wound Healing (C1)”, “IFN-γ Dominant (C2)”, was significantly enriched in the invasive C1 subtype (Fig. 2D). In addition, the TIMER algorithm showed that patients with the C2 subtype had more abundant immune cell infiltration, including infiltration of B cells, CD4 T cells, neutrophils, macrophages, and DCs (Fig. 2E).

### Distribution of mutation frequencies among different invasive subtypes in lung adenocarcinoma

**Figure 3:**
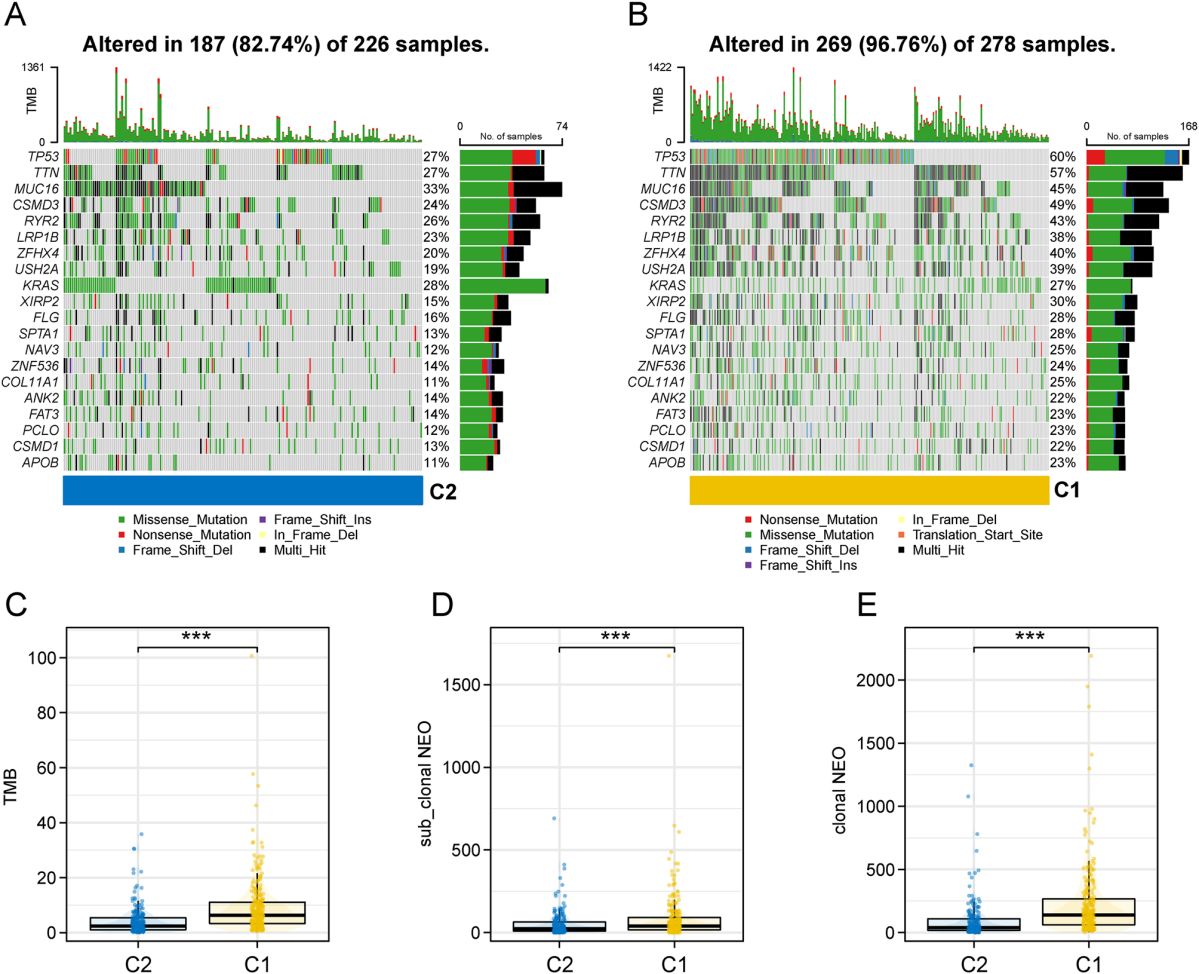
Mutation frequencies of different subtypes (**A**,**B**) Waterfall plot of mutation frequencies of the top 20 genes. (**C**) Differences in TMB distribution among different subtypes. (**D**,**E**) Differences in the distribution of sub-clonal neoantigens and clonal neoantigens in patients with different subtypes. (***p < 0.001; **p < 0.01; *p < 0.05; *ns* not significant).

We further analyzed the gene mutation frequencies to gain further biological insight into the immunological properties of the different invasive LUAD subtypes. Among the 20 genes with the highest mutation frequencies, we found that most genes were significantly more frequently mutated in the C1 subtype than in the C2 subtype. The largest difference was in the TP53 mutation, which was 27% in the C2 subtype and 60% in the C1 subtype (Fig. 3A,B). Next, we analyzed the distribution of tumor mutational load (TMB), clonal neoantigens and sub-clonal neoantigens in different subtypes, and the results showed that TMB, clonal neoantigens and sub-clonal neoantigens were higher in C1 subtypes than in C2 subtypes (Fig. 3C,D,E).

### Identification of hub genes of invasive subtypes in lung adenocarcinoma

**Figure 4:**
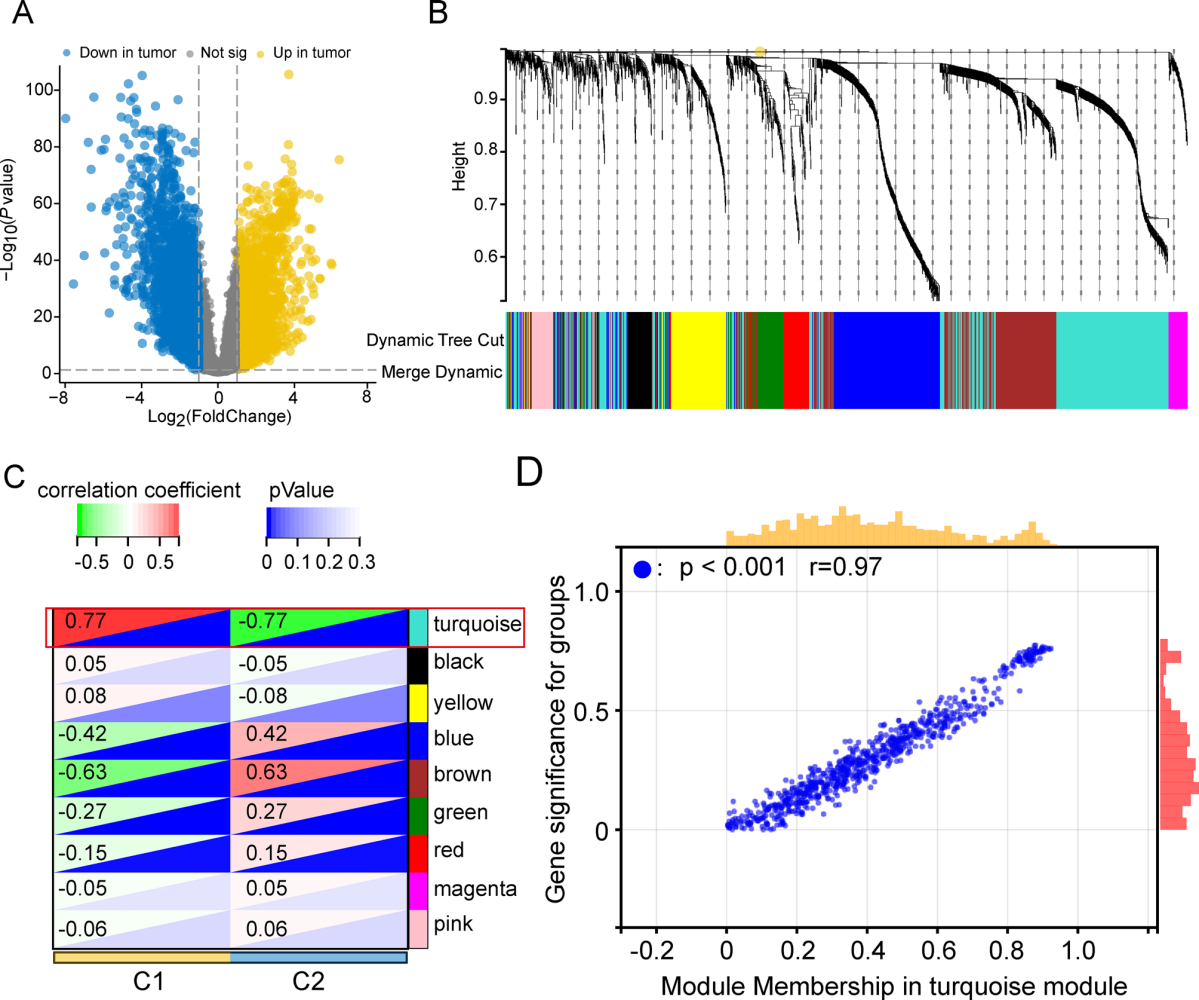
Screening of hub genes with the invasive subtype. (**A**) Analysis of differential genes between tumor and normal tissues. (**B**) Clustering dendrogram of co-expression network modules. (**C**) Correlation analysis of different gene modules with invasive subtypes. (**D**) The Module Membership (MM) versus Gene Significance (GS) scatterplot for C1 subtype in turquoise module. Each dot represents a gene, and the threshold is Module Membership > 0.8 and Gene Significance > 0.6.

To identify potential core genes that can influence the aggressiveness of lung adenocarcinoma. We first obtained 4462 genes with aberrant expression at the tumor site (Fig. 4A) for subsequent WGCNA analysis. Then, we chose β = 3 to construct an unsigned scale-free co-expression network (Supplementary Fig. 3A,B), next we divided these genes into nine co-expressed gene modules (Fig. 4B). Correlation analysis showed that the turquoise module had the highest correlation (r = 0.77, p < 0.001) with the invasive C1 subtype (Fig. 4C). Further 72 core genes associated with the invasive phenotype were screened in the turquoise module using a threshold of MM > 0.8 and GS > 0.6 (Fig. 4D, Supplementary Table 4).

### Construction of an invasive score for lung adenocarcinoma

**Figure 5:**
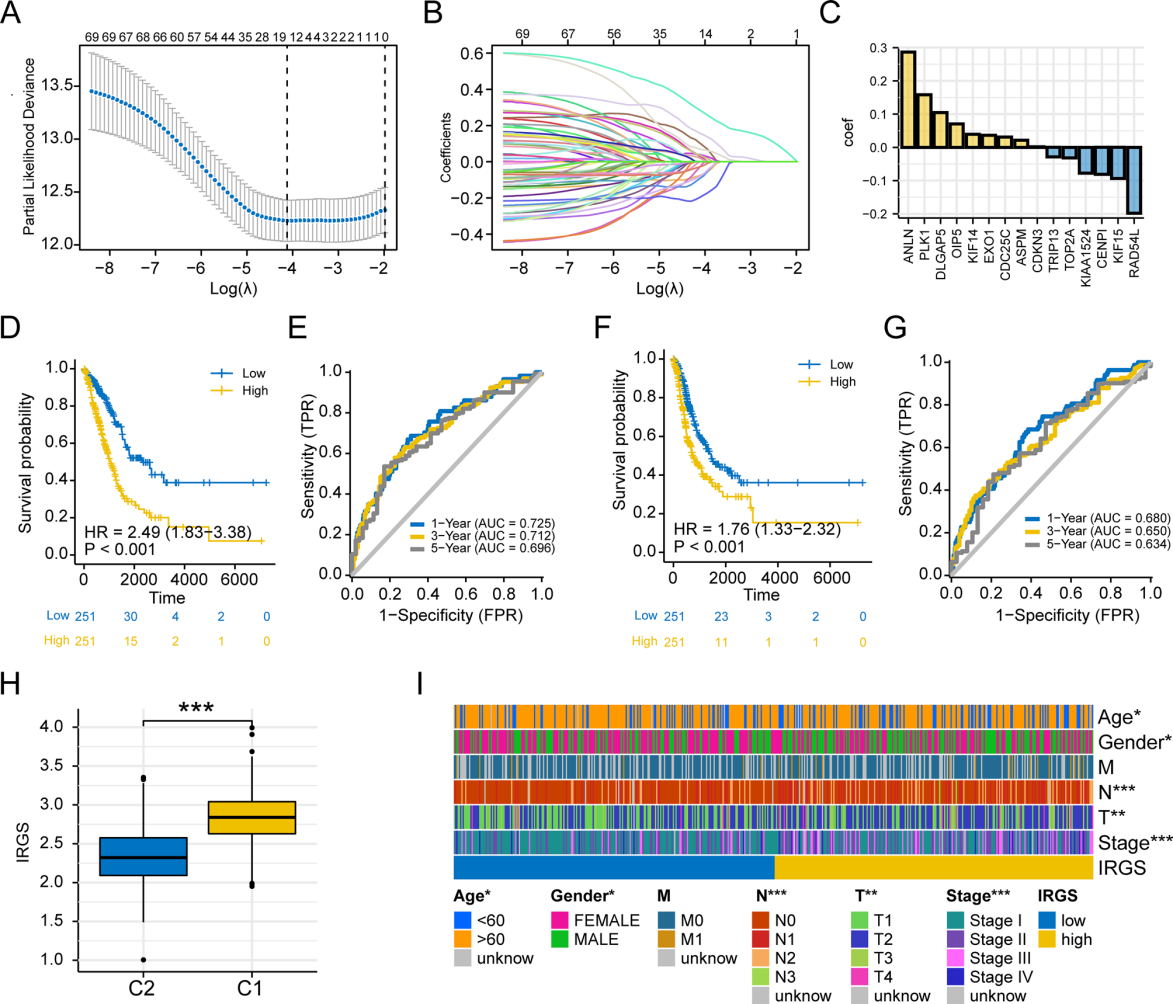
Construction of invasion score in lung adenocarcinoma. (**A**–**C**) LASSO regression analysis to identify 15 prognosis-related and invasion-related core genes and build a risk signature. (**D**,**E**) Efficacy of IRGS in predicting OS in lung adenocarcinoma. (**F**,**G**) Efficacy of IRGS in predicting PFI in lung adenocarcinoma. (**H**) Differences in the distribution of IRGS in C1 and C2 subtypes. (**I**) Heatmap of IRGS distribution with clinicopathological features (***p < 0.001; **p < 0.01; *p < 0.05; *ns* not significant).

LASSO regression analysis was applied to 72 invasive key genes, and 15 genes highly associated with prognosis were finally identified (Fig. 5A–C), based on which the Invasion Related Genes Score (IRGS) was established. IRGS = 0.104698320890061 × DLGAP5 + 0.0705485337432641 × OIP5 − 0.0775757540553236 × KIAA1524 − 0.0317695961025775 × TOP2A + 0.0394790405209059 × KIF14 + 0.0310392983776807 × CDC25C + 0.28657897467505 × ANLN − 0.198017556782708 × RAD54L + 0.0214605988960615 × ASPM + 0.00156005239193903 × CDKN3 − 0.0938154152350938 × KIF15 + 0.158184810717022 × PLK1 + 0.0362675024230202 × EXO1 − 0.0812149879001612 × CENPI − 0.0288045135540301 × TRIP13. Prognostic analysis showed that patients in the low-risk group had significantly better OS than those in the high-risk group (HR 2.49, p < 0.001) (Fig. 5D), while the area under the ROC curve (AUC) of IRGS predicting OS at 1, 3, 5 years was 0.725, 0.712, 0.696 (Fig. 5E), respectively. Similarly, patients in the low-risk group had significantly better PFI than those in the high-risk group (HR 1.76, p < 0.001) (Fig. 5F), and the area under the ROC curve (AUC) for predicting PFI at 1, 3, 5 years was 0.680, 0.650, 0.634 (Fig. 5G), respectively. Notably, the IRGS of patients in the C1 subtype was significantly higher than that in the C2 subtype (Fig. 5H). This result indicated a high correlation between IRGS and the invasive subtype. We then further explored the association between IRGS and the clinicopathological features of LUAD patients. The results showed that IRGS was significantly correlated with age, gender, T-stage, N-stage, and pathological stage (Fig. 5I). Furthermore, univariate and multivariate Cox analysis combined with clinicopathological features showed that IRGS was an independent prognostic factor (Supplementary Fig. 4A,B), and the nomogram constructed based on IRGS also has a good efficiency in predicting 1, 3, 5-year survival of patients (Supplementary Fig. 4C,D).

### Robustness of IRGS in predicting invasive phenotype and prognosis of lung adenocarcinoma

**Figure 6:**
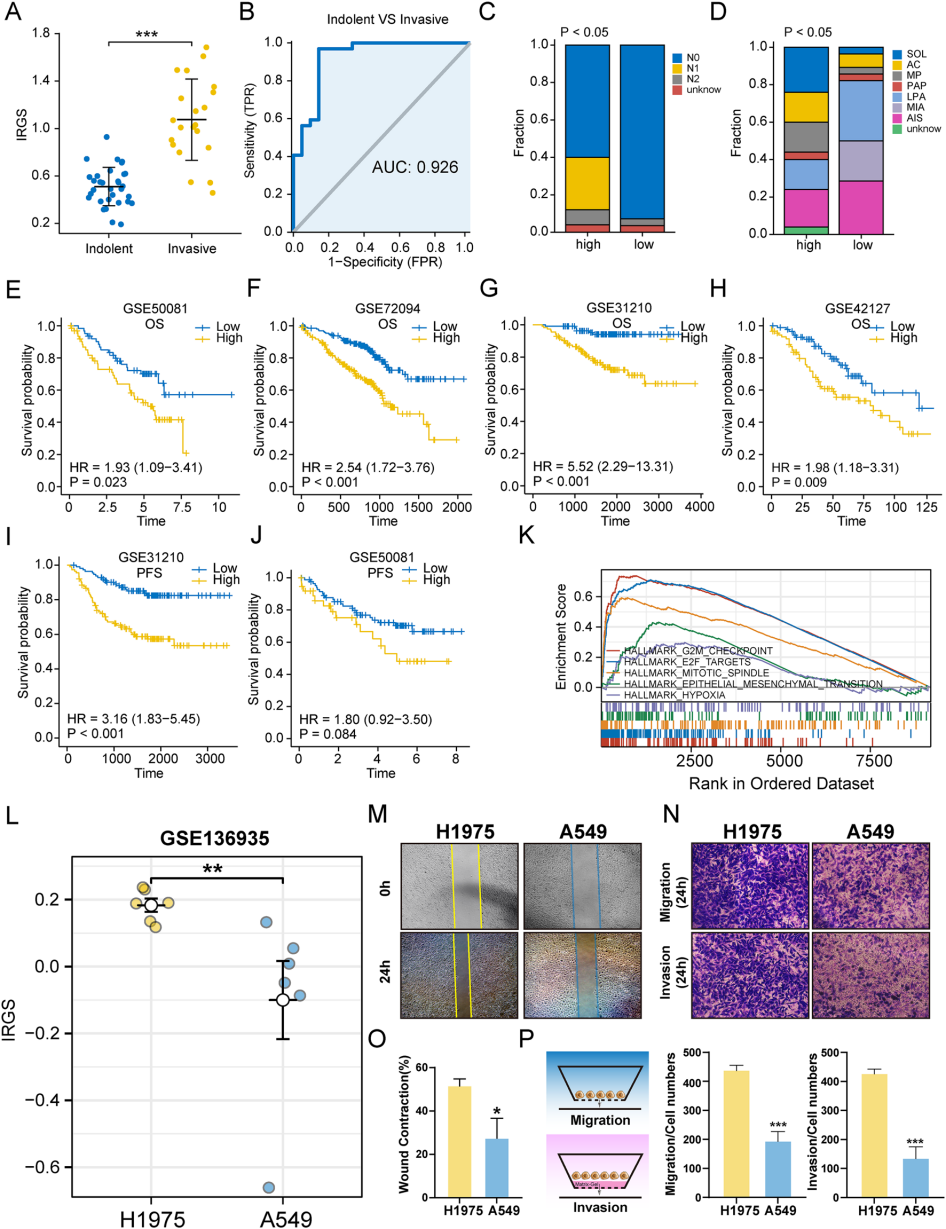
Validation of the predictive power of IRGS (**A**) Differences in the distribution of IRGS between the invasive and indolent groups defined by Yoo et al. (**B**) ROC curves of IRGS in distinguishing invasive from indolent groups. (**C**) Stacked plots of the distribution of different N-stage patients in high and low IRGS groups (Chi-square test). (**D**) Stacked plots of the distribution of different histological subtypes of patients in high and low IRGS groups (Chi-square test). (**E**–**H**) Efficacy of IRGS in predicting OS in independent GEO datasets. (**I**,**J**) Efficacy of IRGS in predicting PFS in independent GEO datasets. (**K**) GSEA analysis of IRGS-related biological pathways. (**L**) Gene expression of A549 and H1975 lung adenocarcinoma cell lines treated with PBS only was extracted from the GSE136935 dataset and IRGS of H1975 and A549 were calculated and compared. (M) Scratch assay to detect the migration ability of H1975 and A549. (**N**) Transwell assay for migration and invasion ability of H1975 and A549. (**O**) Quantitative analysis of scratch assay. (**P**) Schematic diagram and quantitative analysis of transwell assay (***p < 0.001; **p < 0.01; *p < 0.05; *ns* not significant).

To further clarify the efficacy of IRGS in predicting invasive phenotype in LUAD. External validation was performed by the GSE166722 dataset. The results showed that high IRGS was mainly enriched in the invasive group (Fig. 6A), while IRGS reached an AUC of 0.926 in distinguishing indolent and invasive patients (Fig. 6B). The stacked plot showed that there were significantly more patients with N1 and N2 stages in the high-risk group compared to the low-risk group (Fig. 6C); moreover, the invasive histological subtypes SOL, AC, MP, and PAP were enriched in the high-risk group, while the less invasive histological subtypes LPA, MIA, and AIS were enriched in the low-risk group (Fig. 6D). In addition, IRGS was also significantly upregulated in the more invasive TGFBR2-deficient mouse model^32^ (Supplementary Fig. 5A,B). In conclusion, these results suggest that IRGS has good performance in assessing invasive as well as metastatic ability.

We then compared the predictive ability of IRGS for survival in patients with lung adenocarcinoma in different GEO datasets. K-M curves showed that IRGS was highly efficient in predicting survival, GSE50081 (OS: HR 1.93; PFS: HR 1.80), GSE72094 (OS: HR 2.54), GSE31210 (OS: HR 5.52; PFS: HR 3.16), and GSE42127 (OS: HR 1.98) (Fig. 6E–J). In addition, GSEA analysis showed that the high-risk group was enriched in G2M_CHEKPOINT, EMT and other cell proliferation, cell migration-related pathways (Fig. 6K, Supplementary Table 5).

To assess the efficacy of IRGS in predicting invasive ability in vitro. We extracted the gene expression of A549 and H1975 from the GSE136935 dataset and calculated the IRGS. The results showed that the IRGS of H1975 was higher than that of A549 (Fig. 6L). Similarly, the results of the transwell assay and scratch assay also showed that H1975 with higher IRGS showed stronger migration and invasion ability after 24 h incubation (Fig. 6M–P). Furthermore, considering that H1975 is an EGFR mutant cell and A549 is a KRAS mutant cell^33^. This may affect the invasion prediction efficacy of IRGS. So we then compared the invasion ability of H1975 and PC9, which are EGFR mutant cell lines^34^. Transcriptome expression profiles of H1975 and PC9 were obtained from GSE202859 and IRGS was calculated. The results showed that IRGS of H1975 was higher than PC9 (Supplementary Fig. 6A). Subsequent in vitro experiments showed that H1975 had a higher invasive ability compared to PC9 (Supplementary Fig. 6B,C). Overall, these results demonstrated the predictive efficacy of IRGS for invasion.

### The relationship between IRGS and the remodeling of tumor microenvironment in lung adenocarcinoma

**Figure 7:**
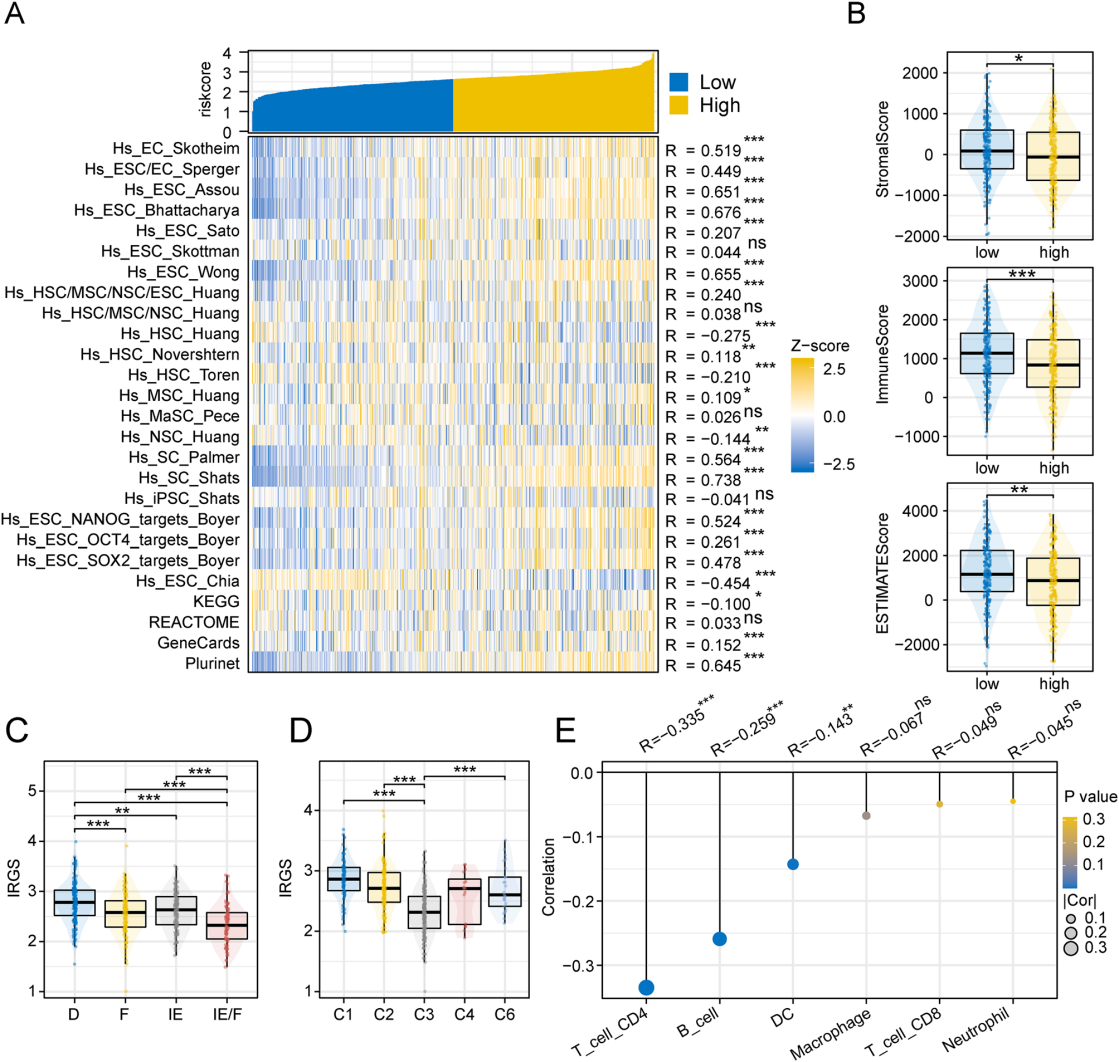
Relevance of IRGS to TME formation. (**A**) Heatmap of co-expression of IRGS with 26 stemness signature scores. (**B**) Differences in immune, stromal, and ESTIMATE scores between high- and low-risk groups. Differences in the distribution of IRGS in (**C**) defined TME subtypes and (**D**) defined immune subtypes. (**E**) TIMER algorithm to calculate the correlation between IRGS and immune cell infiltration using the Spearman test (***p < 0.001; **p < 0.01; *p < 0.05; *ns* not significant).

By calculating the correlations between IRGS and 26 stemness signature scores, we found that as IRGS gradually increased, most stemness characteristic scores also increased, including Hs_EC_Skotheim (R = 0.519), Hs_ESC/EC_Sperger (R = 0.449), Hs_EC_Assou (R = 0.651), Hs_EC_Bhattacharya (R = 0.676) and so on; furthermore, Hs_HSC_Huang (R = -0.275), Hs_HSC_Toren (R = -0.210), Hs_HSC_ Chia (R = − 0.454) and other stemness feature scores were negatively correlated with IRGS (Fig. 7A). The ESTIMATE algorithm showed that in the high-risk group, stromal scores, immune scores, and ESTIMATE scores were downregulated (Fig. 7B). Compared to other defined TME subtypes, IRGS is mainly enriched in the “immune-depleted (D) subtype” (Fig. 7C). Similarly, in terms of immune subtypes, IRGS is least distributed in the “Inflammatory subtype (C3)” and most distributed in the “Wound Healing subtype (C1)” and “IFN-γ Dominant” (C2) (Fig. 7D). The TIMER algorithm showed a significant negative correlation between IRGS and the infiltration abundance of CD4 T cells, B cells and DCs (Fig. 7E). Further, we evaluated IRGS of NSCLC cell lines from the CCLE database (Supplementary Table 6). (Supplementary Fig. 7A) shows the expression of stemness markers, immune checkpoints and IRGS. Subsequently, we evaluated the correlation between IRGS and stemness markers as well as immune checkpoints separately. The results showed that the expression of CD44, CD166, CD90 and CD87 stemness markers in tumor cells increased with the elevation of IRGS (Supplementary Fig. 8A). In terms of immune checkpoint expression pattern, IRGS was positively correlated with common immunosuppressants including TGFB1, CD274 and PDCD1LG2 (Supplementary Fig. 8B). This further suggests a relationship between IRGS and the formation of an immunosuppressive microenvironment.

### Prediction of drug sensitivity and potential therapeutic agents based on IRGS

**Figure 8:**
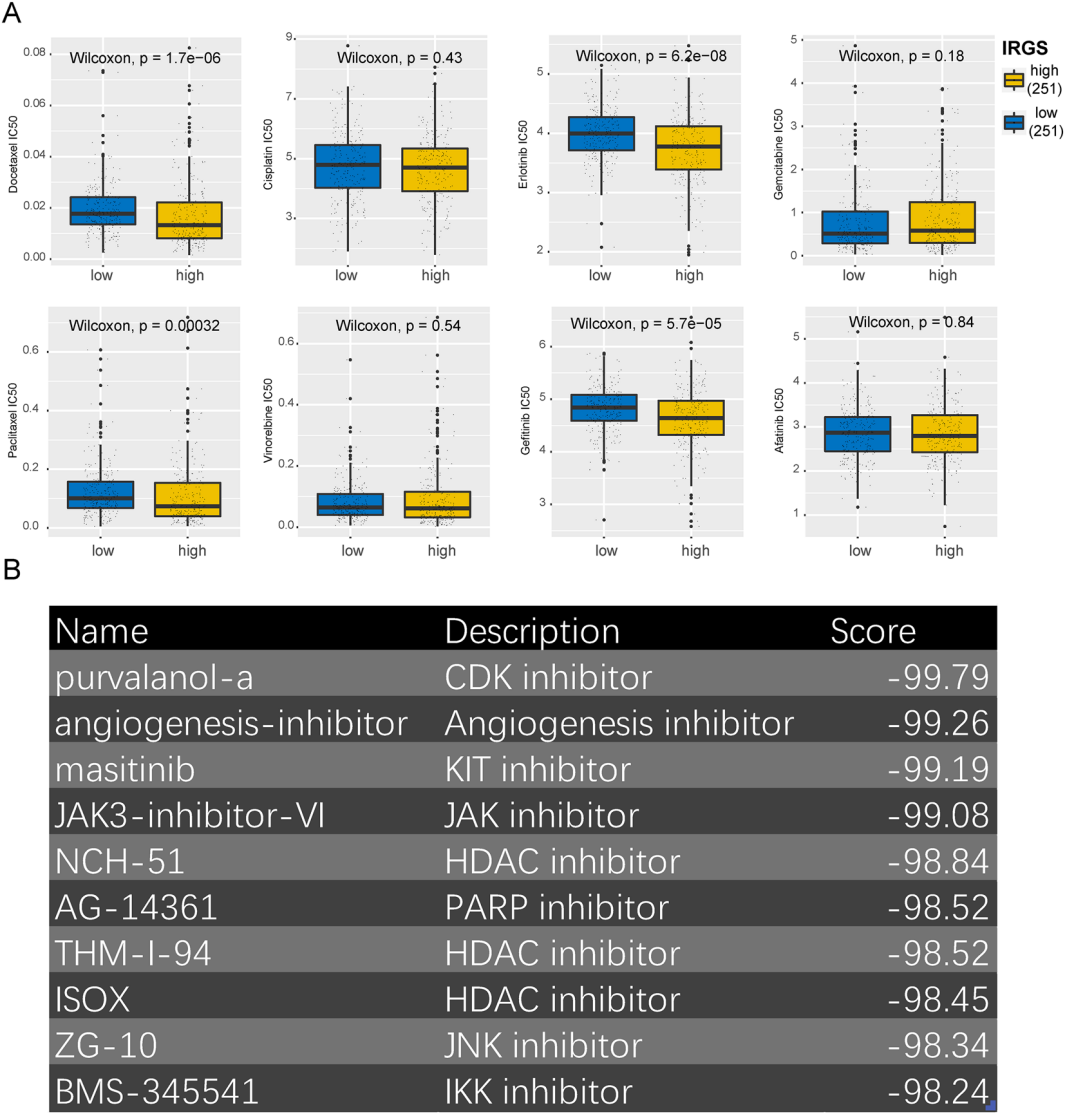
Correlation between IRGS and drug sensitivity. (**A**) Differences in drug sensitivity between different IRGS subgroups. (**B**) Screening of potential therapeutic agents for invasive LUAD based on CMap.

To further explore the potential clinical value of IRGS, we predicted the IC_50_ to common chemotherapeutic agents and targeted therapeutic agents for each LUAD patient using the “oncoPredict” R package, utilizing the IC_50_ concentration as an indicator for assessing drug sensitivity. And based on the median value of IRGS, patients were categorized into high and low IRGS groups. Then, the differences in drug sensitivity between the two groups were compared. The results showed that patients in the high-risk group were more sensitive to Docetaxel, Erlotinib, Paclitaxel, and Gefitinib (Fig. 8A). Subsequently, we grouped the tumor cells into H1975 (high IRGS) and A549 (low IRGS) groups, as well as H1975 (high IRGS) and PC9 (low IRGS) groups, respectively. And we compared the differences in drug sensitivity of these cells to paclitaxel. The results showed that the growth of tumor cells was inhibited after administration of the drug. At 72 h, we found that the inhibitory effect of H1975 was stronger than the other two cells (Supplementary Fig. 9A–D). This suggests the validity of IRGS for assessing drug sensitivity. Next, we attempted to screen the drugs that potentially inhibit tumor invasion and metastasis by IRGS. We screened 110 genes upregulated and 68 genes downregulated in the high-risk group using the criteria of |log_2_FC| > 1.5 and FDR < 0.05 (Supplementary Table 7) and imported them into the Connectivity Map (CMap) database to identify small molecule drugs that might interfere with molecular expression. The results showed that drugs including purvalanol-a, angiogenesis-inhibitor, and masitinib had inhibitory effects on the gene expression profile of patients in the high-risk group (Fig. 8B). Among them, purvalanol-a, a CDK inhibitor, had the highest perturbation score, indicating its potential therapeutic effect in invasive lung adenocarcinoma.

## Discussion

Numerous studies have demonstrated that heterogeneity within the tumor significantly influences the invasive and metastatic capacity of the tumor^35–37^. For example, Yang et al. found that in the early lung adenocarcinoma microenvironment, THBS2^+^CAF could suppress antitumor immunity and promote early lung adenocarcinoma aggressiveness through interaction with B cells and CD8+ T lymphocytes^38^. On the other hand, Caso et al. revealed differences in TMB, APOBEC mutational features, and oncogenic pathway activation among different invasive lung adenocarcinoma pathological histological subtypes by genomic level analysis^39^. Therefore, risk stratification of patients by revealing the differences in invasive molecular features present within lung adenocarcinoma and adopting more targeted treatment strategies may help to improve LUAD patient survival and quality of life.

In this study, we first defined two subtypes of LUAD based on metastasis-related pathway activity. These two subtypes exhibited distinctly different prognostic and clinicopathological features. Among them, the invasive and proliferative genetic features were significantly enriched in the C1 subtype. This suggests a preference for an invasive phenotype in the C1 subtype. Consistently, epithelial-mesenchymal transition (EMT), an important transitional mode of tumor metastasis, was significantly upregulated in the C1 group. In addition, oncogenic pathways such as VEGF, MAPK, PI3K, and Hypoxia were also significantly enriched in the C1 group. These pathways have been shown to play an important role in promoting tumor cell growth and invasion^40–43^. Cancer stem cells (CSC) are important members driving tumor invasion, and past studies have demonstrated through patients and in vivo models that CSC have invasive potential in a variety of cancers and are involved in multiple invasive signaling pathways^44–46^. In our study, the activity of stemness signature genes was significantly upregulated in the C1 group, which further supports the highly invasive properties of the C1 subtype. And in terms of immune infiltration. The different subtypes also showed significant differences, with the C2 type having a richer immune infiltration and tending to be the immune ‘hot’ phenotype, while the C1 type tended to be more immune ‘cold’ phenotype. It is now believed that an active immune response is currently considered to be a favorable factor in improving the survival of cancer patients^47^. Among them, CD4 T cells serve as the core of the body to initiate immune protection, which can inhibit tumor progression by enhancing the tumor-killing activity of anti-tumor effector cells^48^. DC, as the outpost of the host immune response, is a key element in initiating and maintaining the T cell-mediated anti-tumor immune response^49^. Consistently, significant differences in the distribution of defined TME attributes were shown between C1 and C2 subtypes.

To further understand the immunological features between different subtypes. We analyzed the differences in gene mutations between the different subtypes. The difference in mutation frequencies between the two groups was significant. One of the largest mutation frequency differences between groups was in TP53 mutations. As the most common mutational event in multiple cancers, TP53 mutations tend to be associated with a more invasive, more malignant phenotype and poorer survival^50,51^. Here, the attribute of having higher frequency TP53 mutations in the C1 subtype partly explains the clinical outcome of C1 subtypes with an invasive phenotype and poorer survival prognosis.

After comprehensive bioinformatics analysis, we constructed a risk model (IRGS) to quantify the invasiveness ability of LUAD. IRGS was mainly enriched in C1 subtype, which was mainly composed of DLGAP5, OIP5, KIAA1524, TOP2A, KIF14, CDC25C, ANLN, RAD54L, ASPM, CDKN3 KIF15, PLK1, EXO1, CENPI and TRIP13. Among them, DLGAP5, a mitotic spindle protein, has been reported to play an important role in the diagnosis, prognosis, and metastasis of lung cancer^52,53^. OIP5 encodes a protein associated with cancer/testis antigen (CTA) and has been shown to promote oncogenic signaling by interacting with the mTORC1 and β-linked protein pathways^54^. ANLN is an actin-binding protein, and related studies have confirmed that ANLN can affect lung adenocarcinoma progression by promoting epithelial mesenchymalization^55^. PLK1 can promote cancer growth by inhibiting the function of P53, which leads to decreased survival of patients^56^. In summary, these genes have been shown in past studies to significantly correlate with the more invasive and more malignant features of tumors.

The TCGA and GEO cohorts consistently demonstrated that IRGS efficiently predicted survival in patients with LUAD. Also, patients with high IRGS showed poor clinic pathological parameters. After adjusting for other confounding factors, IRGS remained an independent predictor of OS in LUAD patients. In a previous report, Yoo et al. effectively divided LUAD patients into invasive and indolent groups by 1322 invasive signature genes^16^. Our study showed that IRGS was significantly upregulated in the invasive group, while the invasive LUAD histological subtype was also enriched in patients with high levels of IRGS. Notably, previous studies have shown aberrant expression of oncogenic signaling pathways related to cell growth and cell proliferation, including p53, within the aggressive MP/SOL subtype, as well as significantly higher chromosomal instability in the MP/SOL subtype than in other subtypes, which may partly explain the high mutation gene frequency and poorer survival outcome in the C1 group^39^. In addition, TGFBR2-deficient LUAD mouse models are considered to be more prone to lymph node metastasis and poorer survival^32^. Our study showed that IRGS was significantly upregulated in TGFBR2-deficient mice compared to TGFBR2 wild-type mice. Further, H1975 and PC9 are epidermal growth factor receptor (EGFR) mutant cell lines. Previous studies have shown that EGFR mutations induce signaling pathways associated with carcinogenesis, such as PI3K/AKT/mTOR, RAS/RAF/MAPK, and JAK/STAT, and promote malignant phenotypes such as proliferation, survival, angiogenesis, invasion, and metastasis of tumor cells^57–59^. A549 cell is a KRAS mutant cell line, and the signaling dysregulation caused by KRAS mutations includes MET overexpression, PI3K/AKT/mTOR, and RAF/MEK pathways^60^. Overall, similar to the intratumor heterogeneity that exists between different individuals with lung adenocarcinomas, the molecular heterogeneity that exists within these cell lines allows for varying invasive capabilities among each other. Therefore, effective prediction of the invasive capacity of cell lines would be a powerful means of validating the efficiency of IRGS. In the present study, we found that H1975 had higher IRGS compared to A549 and PC9; subsequent in vitro experiments confirmed the greater invasive capacity of H1975. This demonstrated the powerful efficacy of IRGS in predicting the invasive ability of tumor cells.

With regard to TME, there is a high positive correlation between IRGS and most stemness signature scores and stemness markers, and patients with high IRGS are more inclined to the immune ‘cold’ phenotype of TME. In addition, the strong correlation shown between IRGS and immune checkpoints on tumor cells also suggests that IRGS can induce immune escape. It is worth mentioning that we found inconsistencies between the invasion-related subtypes (Fig. 2E) and IRGS (Fig. 7E) in assessing neutrophil and macrophage expression patterns. It is possible that we favored the efficiency of IRGS in predicting prognosis when screening and developing it. This may make the derived IRGS more efficient in predicting prognosis than the original invasive attribute-related subtypes; however, it inevitably loses some efficiency in predicting some features related to the subtypes, such as TME attributes. Moreover, we also found that IRGS were mainly distributed in the C1 and C2 immune subtypes defined by Thorsson et al. and relatively least distributed in the C3 immune subtype. Among them, the C1 immune subtype was characterized by elevated angiogenic gene expression and high proliferation rate, while the C2 immune subtype was dominated by high CD8 signaling, rich TCR diversity as well as high tumor proliferation and invasiveness^23^. Similarly, in a study by Santisteban et al.^61^, it was found that CD8 T cells can induce EMT transformation to promote cancer progression. (in which EMT, as one of the tumor markers, is closely related to the invasive ability of the tumor). In addition, it has also been reported that the remodeling of inflammatory tumor microenvironment in lung adenocarcinoma is closely associated with the altered EMT status^62^. Comparatively, in the C3 subtype, which has the least distribution of IRGS, the TME attributes are dominated by Th17 and Th1, and low to moderate tumor cell proliferation, where Th17 is thought to suppress tumor^63^. From these results, we can see that immune infiltration, including T cells, can induce changes in the invasive capacity of tumors, and that the changes in tumor invasive capacity induced by differences in the type of T-cell infiltration as well as the different degree of infiltration. Overall, multiple factors within TME can contribute to a patient’s transformation to an invasive malignant phenotype.

To further expand the potential clinical value of IRGS. We analyzed the drug sensitivity of each patient, and LUAD patients with high IRGS showed sensitivity to drugs such as Docetaxel, Erlotinib, Paclitaxel, and Gefitinib. Subsequent in vitro experiments validated the greater killing effect of paclitaxel on high IRGS cell lines. This means that this IRGS may be able to provide some extent of guidance in the selection of clinical treatment options. CMap analysis screened for drugs including purvalanol-a, angiogenesis-inhibitor, and masitinib as therapeutic candidates for invasive LUAD. These drugs may have an important role in suppressing the invasive phenotype and preventing metastasis in LUAD. Among them, the CMap database perturbation scores showed that purvalanol-a was the most perturbative drug on the expression of highly aggressive LUAD molecules. purvalanol-a acts as a CDK inhibitor, which effectively inhibits cell progression from the G2 phase to mitosis. In a previous study, Chen et al. reported that purvalanol-a could enhance the cytotoxic effect of purvalanol on non-small cell carcinoma by inhibiting tumor protein 18 (Oncoprotein 18)^64^. In gastric cancer, Iizuka et al. found that purvalanol-a could promote apoptosis in X-ray irradiated gastric cancer cells by activating the active fragment of caspase 3^65^. And in colon cancer, purvalanol-a can promote apoptosis of colon cancer cells by upregulating the protein expression of Bax and Puma^66^. Overall, these studies consistently suggest that purvalanol-a could be a potential therapeutic agent for patients with highly invasive phenotypes, which provides further evidence for purvalanol-a-related clinical drug development.

However, there are still limitations to this study. Firstly, although our invasion-related gene score has been validated in several datasets as well as in vitro experiments in predicting the invasive ability and prognosis of patients. But further in vivo experiments are still needed for validation. Second, Further exploration of the potential link between TME and tumor invasive capacity is still needed to shed more light on the potential factors that contribute to the heterogeneity of tumor invasive capacity. Thirdly, the effects of potential drugs screened based on IRGS for invasive LUAD still need further vivo experimental validation. Furthermore, more clinical samples are still needed to corroborate the efficiency of IRGS in predicting the invasive ability of LUAD patients.

## Conclusions

In summary, this study identified novel invasive molecular subtypes of LUAD based on the expression patterns of metastasis-related pathways and established the invasion-related gene score (IRGS), which is effective in predicting the prognosis and invasiveness of LUAD. It can provide some reference for the selection of clinical decisions.

### Supplementary Information

Supplementary Figures.

Supplementary Legends.

Supplementary Tables.

## Data Availability

The datasets analyzed during the current study are available on the UCSC website (https://xenabrowser.net/datapages/); MSigDB website (http://www.gseamsigdb.org/gsea/msigdb/index.jsp); CCLE database (https://sites.broadinstitute.org/ccle/) and Gene Expression Omnibus (https://www.ncbi.nlm.nih.gov/geo/), including GSE72094 (https://www.ncbi.nlm.nih.gov/geo/query/acc.cgi?acc=GSE72094), GSE31210 (https://www.ncbi.nlm.nih.gov/geo/query/acc.cgi?acc=GSE31210), GSE50081 (https://www.ncbi.nlm.nih.gov/geo/query/acc.cgi?acc=GSE50081), GSE42127 (https://www.ncbi.nlm.nih.gov/geo/query/acc.cgi?acc=GSE42127), GSE166722 (https://www.ncbi.nlm.nih.gov/geo/query/acc.cgi?acc=GSE166722), GSE27717 (https://www.ncbi.nlm.nih.gov/geo/query/acc.cgi?acc=GSE27717), GSE202859 (https://www.ncbi.nlm.nih.gov/geo/query/acc.cgi?acc=GSE202859) and GSE136935 (https://www.ncbi.nlm.nih.gov/geo/query/acc.cgi?acc=GSE136935) datasets.
