# Supplementary material for: Development of an invasion score based on metastasis-related pathway activity profiles for identifying invasive molecular subtypes of lung adenocarcinoma
Source: Sci Rep. 2024 Jan 19;14:1692. doi: 10.1038/s41598-024-51681-9 (PMC10799059; doi:10.1038/s41598-024-51681-9)
Supplement: Supplementary file 1 — Supplementary Figures. [file 41598_2024_51681_MOESM1_ESM.docx]

**Supplementary Figure**


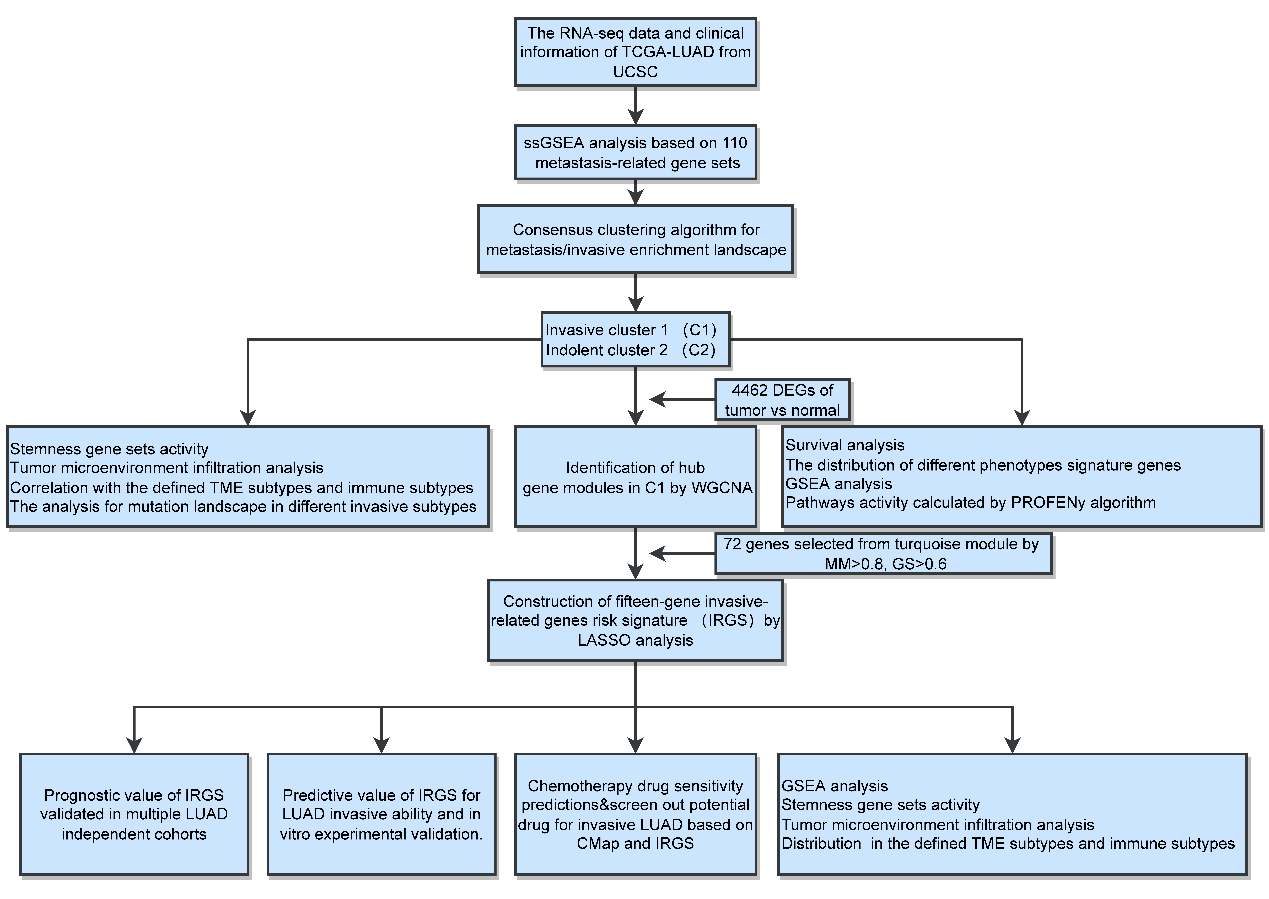


**Supplement Fig.1 Flow chart of the study.**


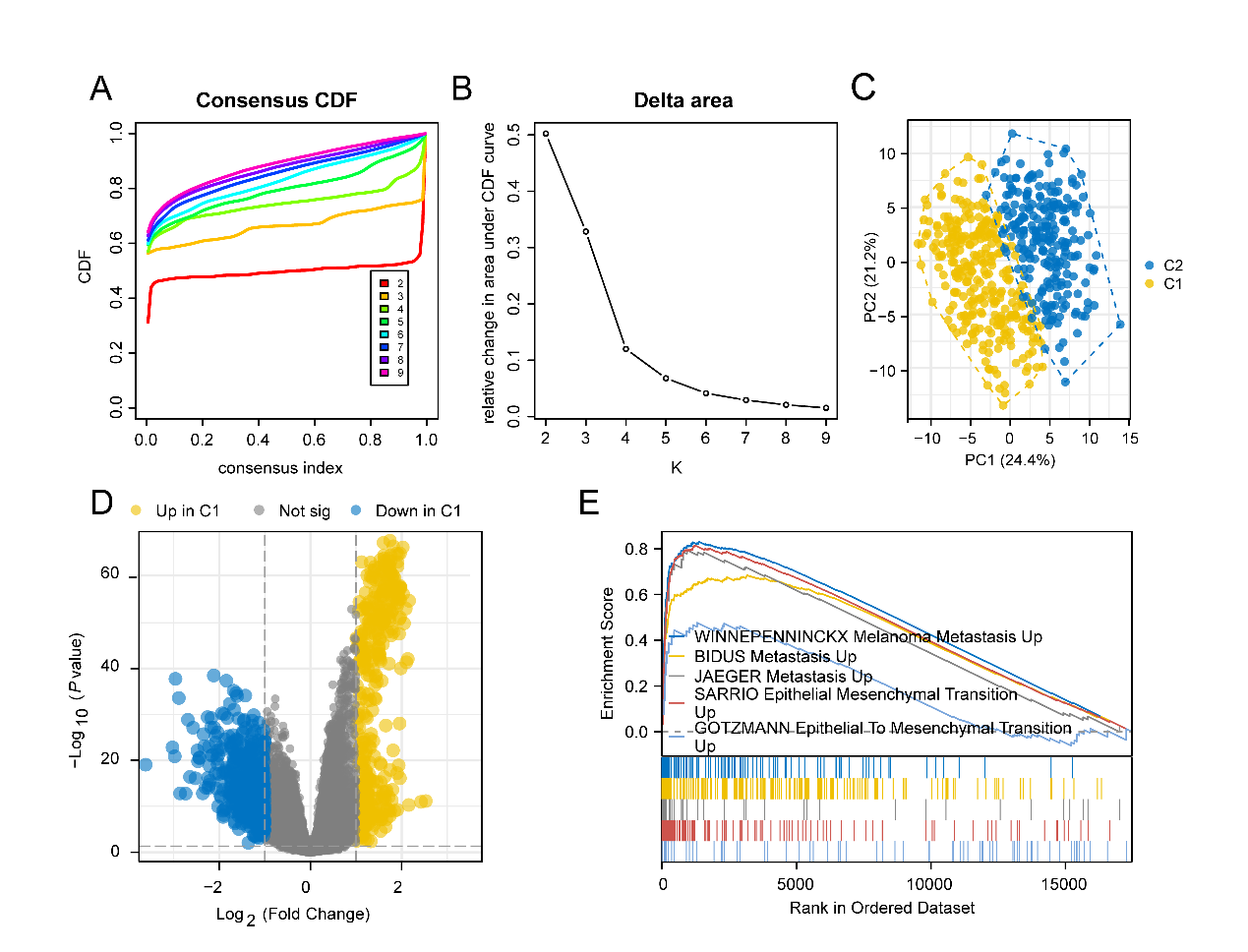


**Supplement Fig.2 Identification of the invasive LUAD subtypes.** (A-B) Delta area curve of consensus clustering. (C) Principal component analysis plots. (D) Volcano plot of gene expression differences between different subtypes. (E) GSEA analysis showing pathways upregulated in C1 subtypes.


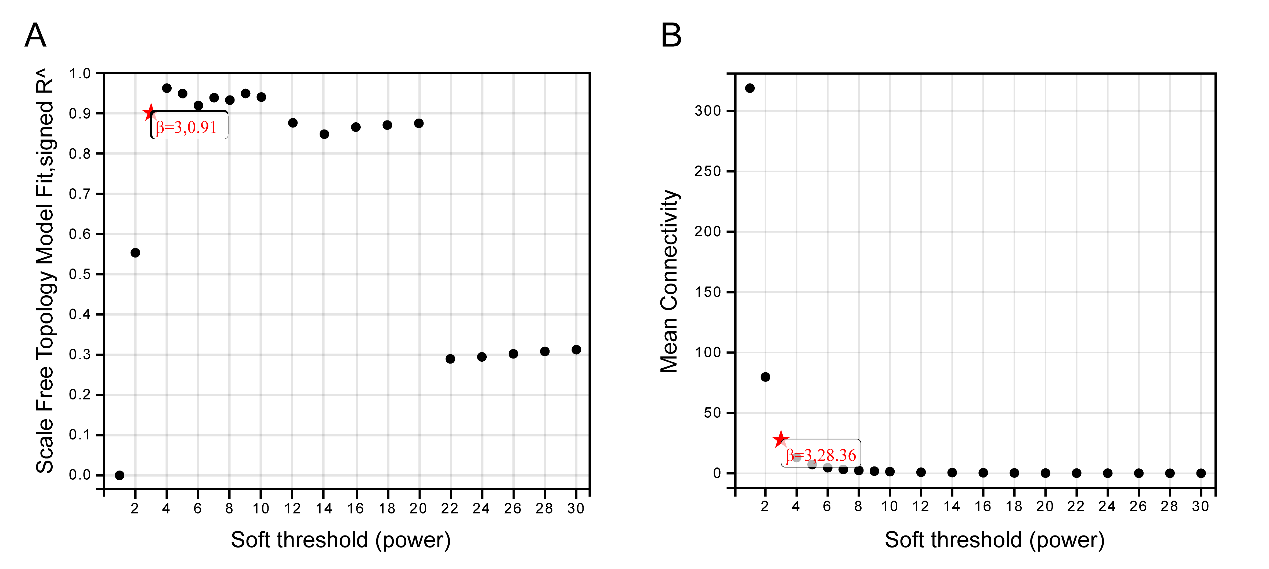


**Supplement Fig.3 The construction of gene co-expression network.** (A) Analyze the scale-free fit index of the 1-30 soft threshold power (β). (B) Analyze the average connectivity of 1-30 soft threshold power.


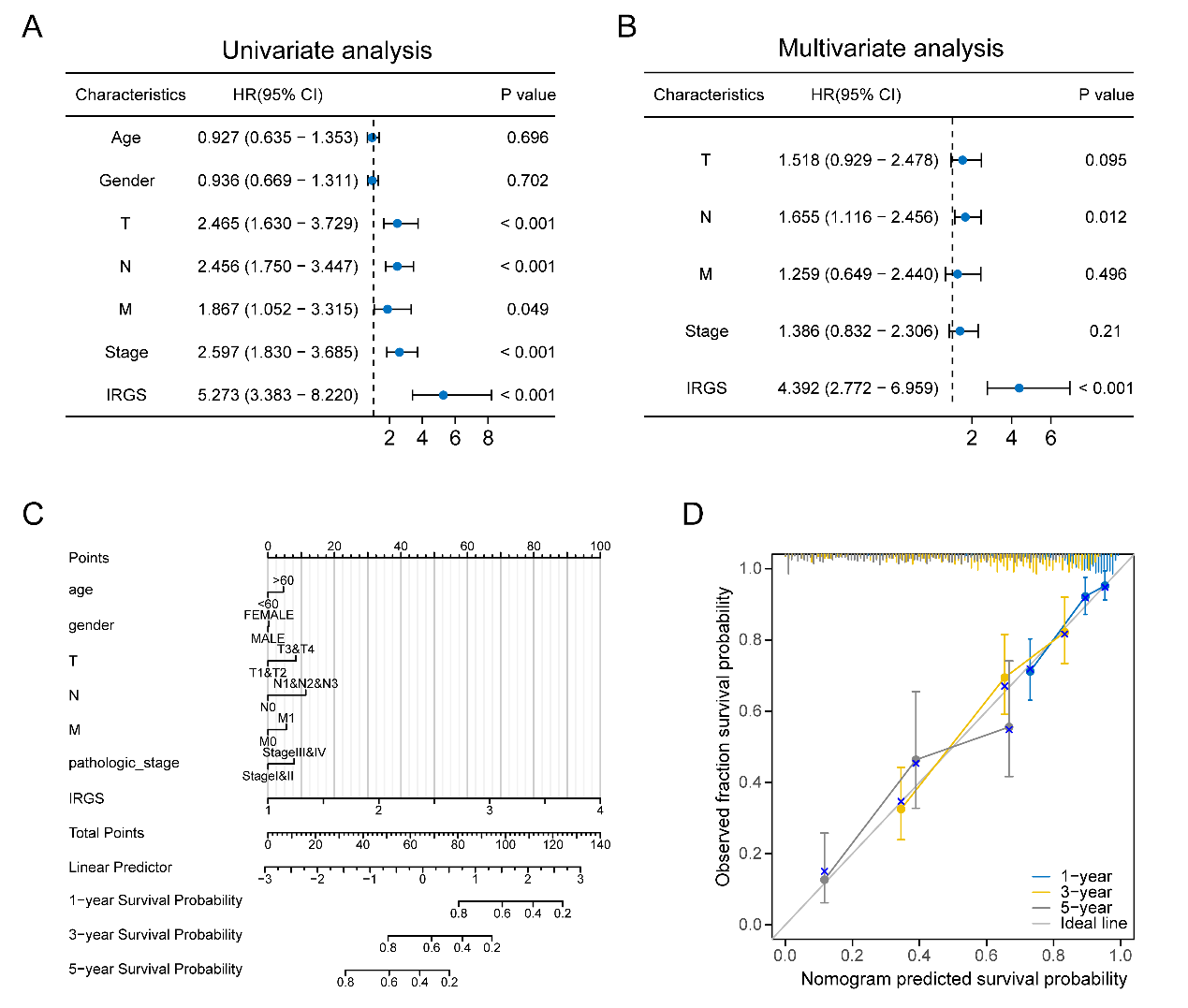


**Supplement Fig.4 The prognostic value of IRGS in LUAD.** (A-B) Univariate and multivariate Cox regression analyses of the association between clinic pathological features and OS of LUAD patients. (C) Construction of a nomogram for survival prediction. (D) The calibration curve for the nomogram model. Three colored lines (blue, yellow, and purple) represent the predicted performance of the nomogram. A closer fit to the diagonal gray line indicates a better effect.


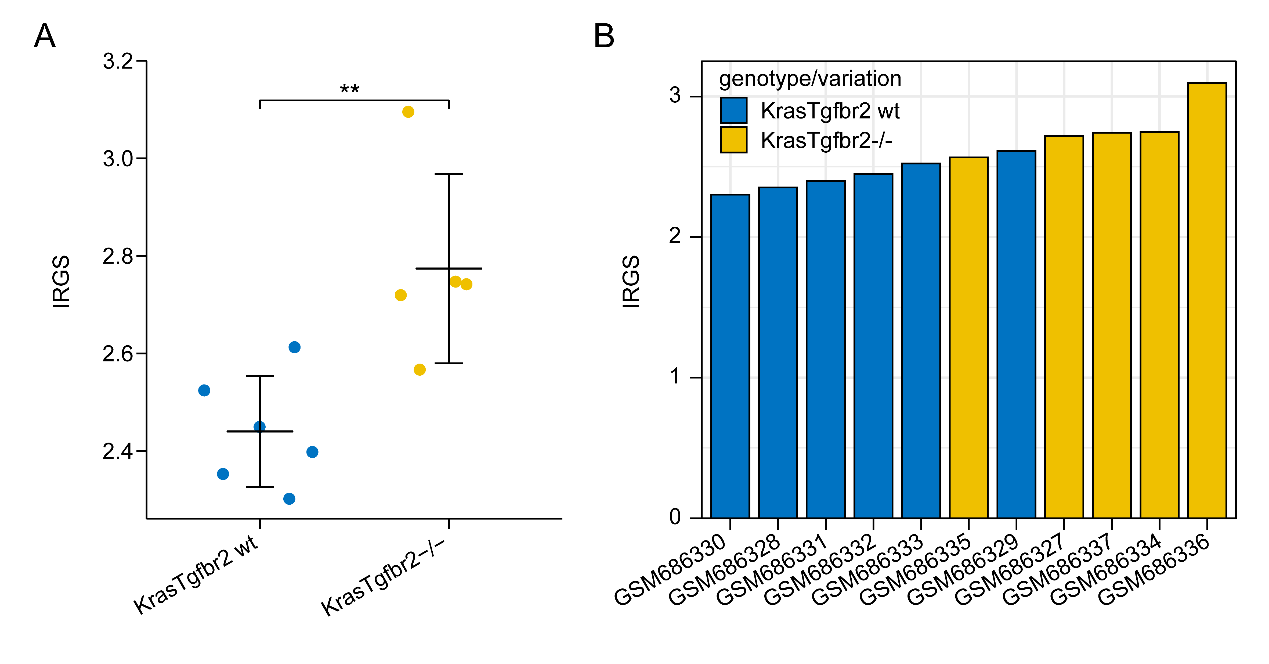


**Supplement Fig.5 Robustness of IRGS in predicting invasiveness in mice model.** (A) Box plot of the difference in the distribution of IRGS in wild-type and TGFBR2-deficient mice. (B) Waterfall plot of the distribution of IRGS in wild-type and TGFBR2-deficient mice.


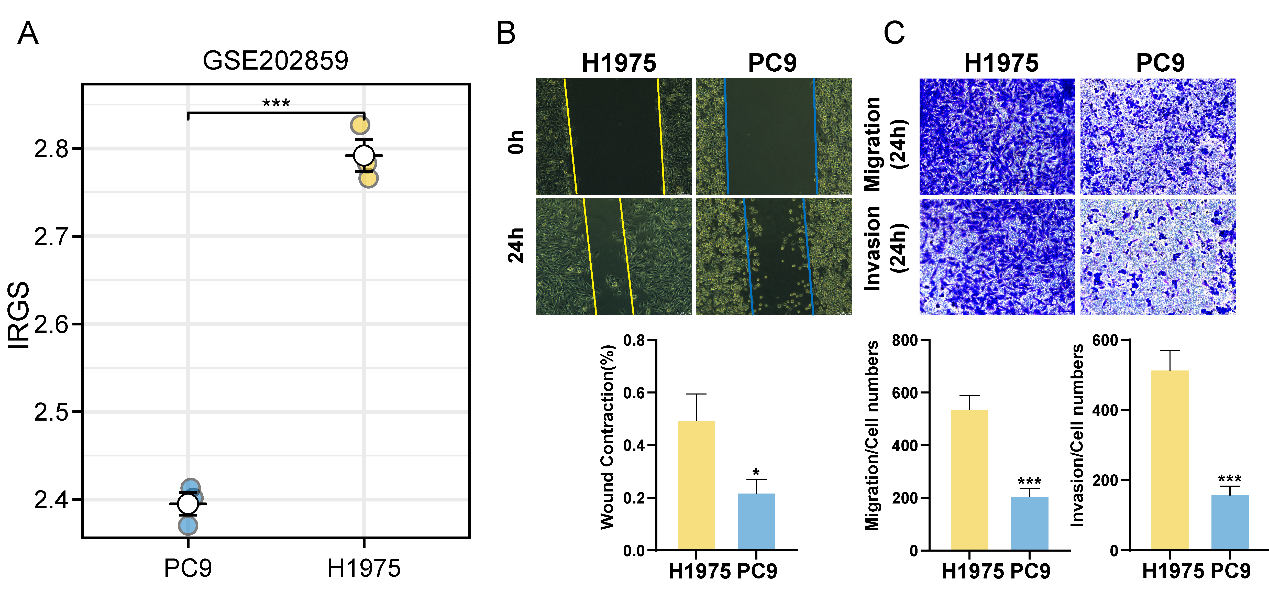


**Supplement Fig.6 Robustness of IRGS in predicting invasiveness in cell lines.** (A) Box plot of the difference in the distribution of IRGS in PC9 and H1975. (B) Scratch assay for the migration ability of H1975 and PC9. (C) Transwell assay for migration and invasion ability of H1975 and PC9. (***p < 0.001; **p < 0.01; *p < 0.05; ns: not significant).


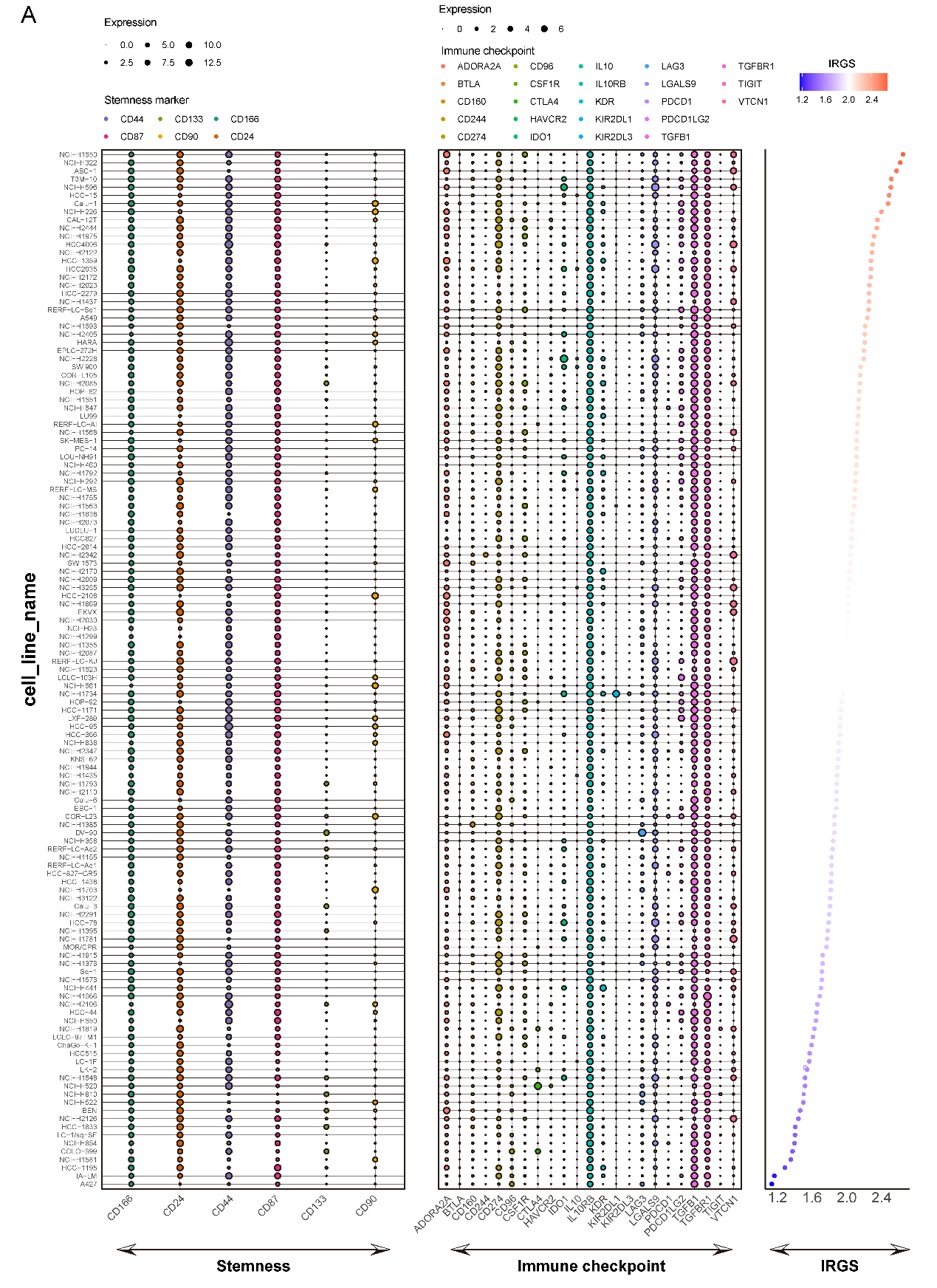


**Supplement Fig.7 IRGS association with stemness marker and immune checkpoints in NSCLC cell lines.** (A) Dot plot of expression profiles of stemness markers with rising IRGS in the cell line; dot plot of expression profiles of the immune checkpoints with rising IRGS in the cell line; dot plot of IRGS in the cell line.


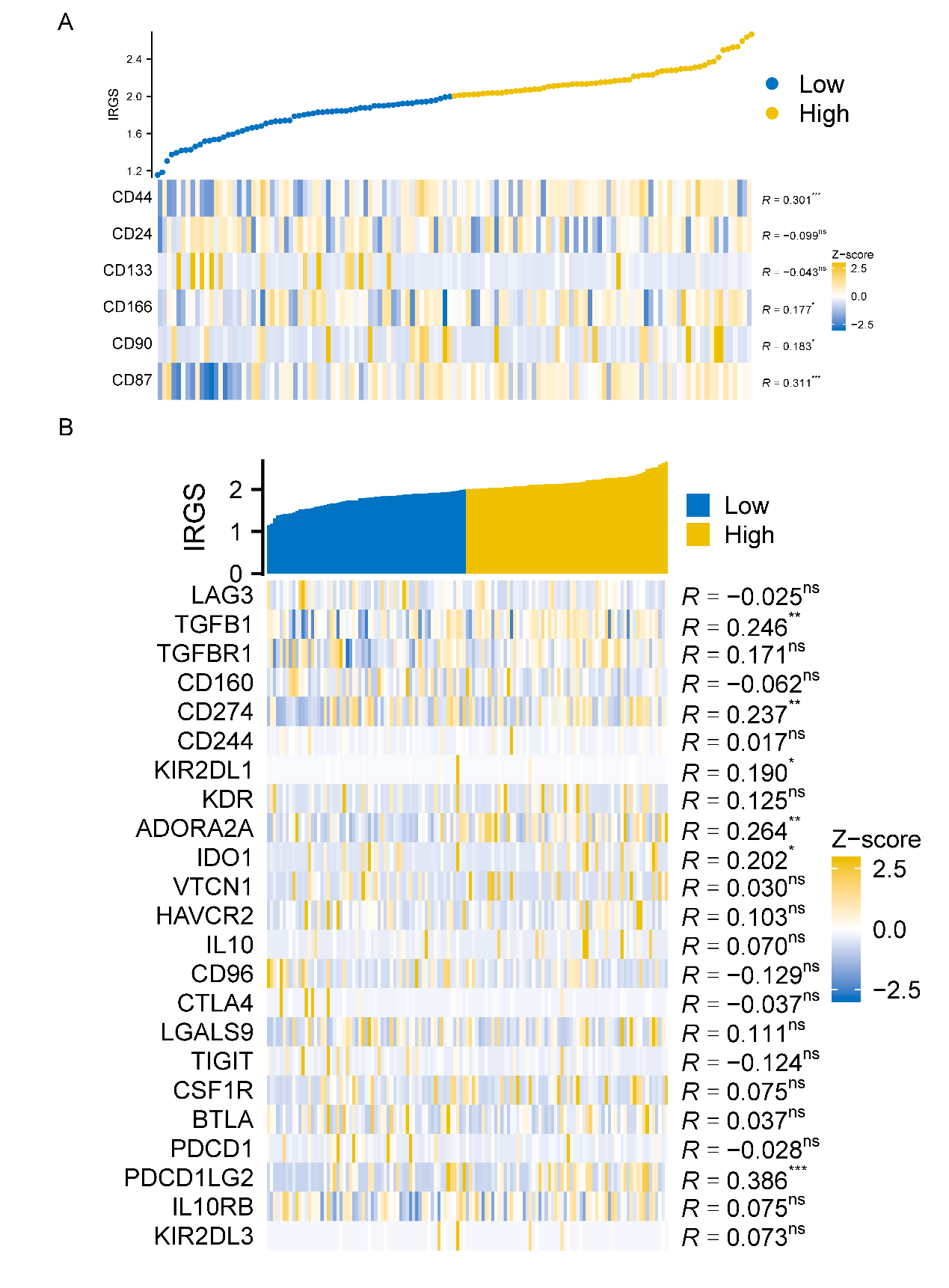


**Supplement Fig.8 Correlation of IRGS with stemness marker and immune checkpoints** (A) Heatmap of IRGS correlation with stemness markers. (B) Heatmap of IRGS correlation with immune checkpoints.


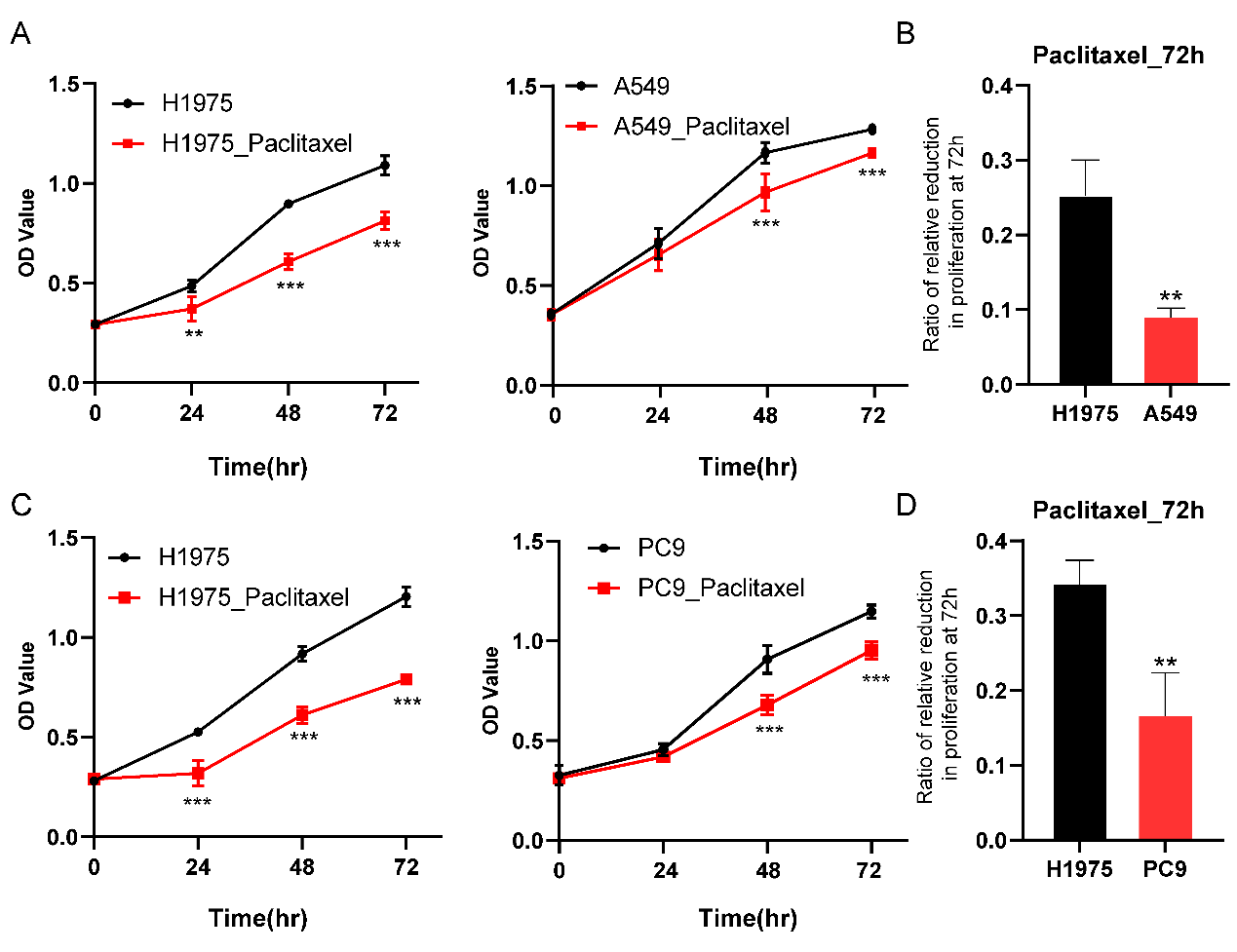


**Supplement Fig.9 Validation of the candidate drug.** (A) The proliferation of control and Paclitaxel (2μM) treated H1975 and A549 cells was measured by MTS assay at the indicated time points. (B) The difference in the reduction of proliferation for Paclitaxel((2μM)) treated H1975 and A549 cells at 72h, Ratio of relative reduction in proliferation = (OD Value (con)- OD Value (Paclitaxel))/OD Value (con). (C) The proliferation of control and Paclitaxel (2μM) treated H1975 and PC9 cells was measured by MTS assay at the indicated time points. (D) The difference in the reduction of proliferation for Paclitaxel((2μM)) treated H1975 and PC9 cells at 72h.
