# Supplementary material for: Development of an invasion score based on metastasis-related pathway activity profiles for identifying invasive molecular subtypes of lung adenocarcinoma
Source: Sci Rep. 2024 Jan 19;14:1692. doi: 10.1038/s41598-024-51681-9 (PMC10799059; doi:10.1038/s41598-024-51681-9)
Supplement: Supplementary file 2 — Supplementary Legends. [file 41598_2024_51681_MOESM2_ESM.docx]

**Table legends**

**Supplementary Table.1:** The dataset numbers and links used for analysis in this article.

**Supplementary Table.2:** The List of metastasis-related gene sets obtained from The Molecular Signatures Database (MSigDB).

**Supplementary Table.3:** Metastatic active gene set scores for TCGA-LUAD.

**Supplementary Table.4:** List of key genes associated with C1 invasive subtypes screened based on WGCNA analysis.

**Supplementary Table.5:** Enrichment level of hallmark gene sets in high IRGS groups.

**Supplementary Table.6:** The IRGS of NSCLC cell lines.

**Supplementary Table.7:** The Key differential genes screened in low and high IRGS groups using the criteria of |log2FC| >1.5 and FDR < 0.05.
